# Silencing a sugar transporter gene reduces growth and fecundity in the brown planthopper, *Nilaparvata lugens* (Stål) (Hemiptera: Delphacidae)

**DOI:** 10.1038/srep12194

**Published:** 2015-07-17

**Authors:** Lin-Quan Ge, Yi-Ping Jiang, Ting Xia, Qi-Sheng Song, David Stanley, Peng Kuai, Xiu-Li Lu, Guo-Qing Yang, Jin-Cai Wu

**Affiliations:** 1 School of Plant Protection Yangzhou University, Yangzhou 225009, P.R. China; 2 Division of Plant Sciences, University of Missouri, 1-31 Agriculture Building, Columbia, MO 65211, USA; 3 USDA/Agricultural Research Service, Biological Control of Insects Research Laboratory, Columbia, Missouri.

## Abstract

The brown planthopper (BPH), *Nilaparvata lugens,* sugar transporter gene 6 (*Nlst6*) is a facilitative glucose/fructose transporter (often called a passive carrier) expressed in midgut that mediates sugar transport from the midgut lumen to hemolymph. The influence of down regulating expression of sugar transporter genes on insect growth, development, and fecundity is unknown. Nonetheless, it is reasonable to suspect that transporter-mediated uptake of dietary sugar is essential to the biology of phloem-feeding insects. Based on this reasoning, we posed the hypothesis that silencing, or reducing expression, of a BPH sugar transporter gene would be deleterious to the insects. To test our hypothesis, we examined the effects of *Nlst6* knockdown on BPH biology. Reducing expression of *Nlst6* led to profound effects on BPHs. It significantly prolonged the pre-oviposition period, shortened the oviposition period, decreased the number of eggs deposited and reduced body weight, compared to controls. *Nlst6* knockdown also significantly decreased fat body and ovarian (particularly vitellogenin) protein content as well as vitellogenin gene expression. Experimental BPHs accumulated less fat body glucose compared to controls. We infer that *Nlst6* acts in BPH growth and fecundity, and has potential as a novel target gene for control of phloem-feeding pest insects.

Hemipteran phloem feeding insects cause persistent yield reductions in many globally important crop species^1^. The brown planthopper (BPH), *Nilaparvata lugens,* is the most serious insect rice pest throughout Asia, with outbreaks increasing in intensity^1^,^2^. BPHs damage rice crops by removing sugars, reducing photosynthesis, blocking phloem elements^3^ and transmitting plant viruses^4^. Control of BPH infestation has depended on deploying broad-spectrum insecticides, however, resistance to commonly used organophosphorous, carbamate and pyrethroid insecticides is now widespread^5^,^6^. The global importance of rice, estimated to supply 20% of the world’s calorific intake, drives research designed to develop new BPH control measures.

Sucrose is the principal carbohydrate in rice phloem and the major energy source for phloem feeders, including the BPH^7^. As a disaccharide, sucrose cannot be transported directly across the hemipteran gut epithelium. It is hydrolyzed into two monosaccharides, glucose and fructose, in the BPH midgut for transport into hemolymph circulation^8^. Sugar transporters act in carbohydrate transport in most organisms, from bacteria to mammals, and are responsible for mediating the movement of sugar into cells^9^. The BPH glucose transporter, *N. lugens* hexose transporter 1 (NlHT1), facilitates the uptake of glucose, but not fructose, in the midgut^10^. BPHs also express a midgut sugar transporter gene, *Nlst6*, that encodes a facilitative glucose/fructose transporter^11^. The aphid, *Acyrthosiphon pisum,* expresses at least two sugar transporters, Ap-ST3 and Ap-ST4, both of which transport glucose and fructose^12^,^13^. In mammals, some glucose transporter (GLUT; e.g., GLUT4) proteins are retained within muscle and adipose cells; insulin signaling leads to increased GLUT trafficking, rapid translocation to cell surfaces and increased glucose uptake. A similar mechanism probably occurs in insects, as well. Transgenic *Drosophila* expressing human GLUT4 in fat body responded to insulin treatments with increased GLUT4 translocation to the surface of fat body cells^14^. GLUT physiology goes beyond sugar transport, however, because GLUT4 also transports dehydroascorbic acid and glucosamine^15^. We presume sugar transporters act in multiple actions in membrane transport and energy acquisition. The genes encoding these transporters may serve as potential targets for RNAi based control of insect pests in transgenic plants. We posed this idea as the hypothesis that silencing these genes will lethally reduce BPH fitness in agroecosystems. Here we report on the outcomes of experiments designed to test our hypothesis.

## Results

### Dietary ds*Nlst6* reduced gene expression

**Figure 1:**
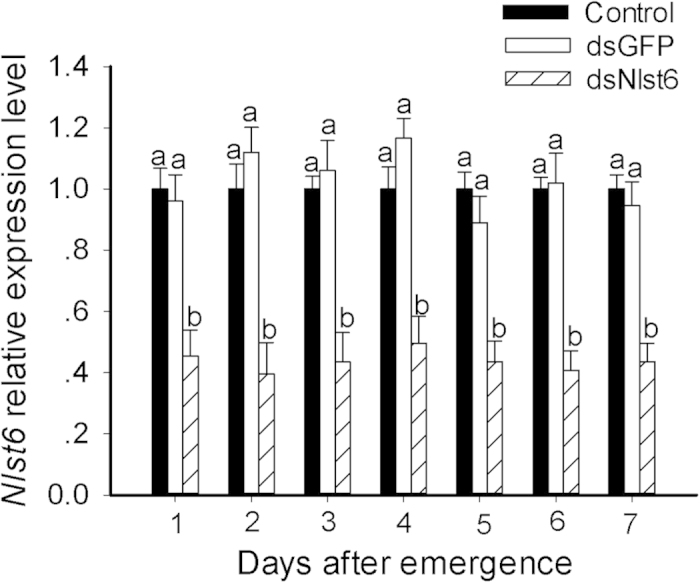
Dietary ds*Nlst6* treatments reduced *Nlst6* expression. All values were normalized relative to β-actin transcript levels. The data represent the mean values ± SE of three independent biological replicates. Histogram bars annotated with the same letter are not significantly different between control and ds*Nlst6* treatments (t-test, *p* < 0.05).

The dietary treatments (for 8 days) did not influence survival of the synchronous nymph groups, at approximately 78% for the experimental group on media amended with 0.05 μg/μl *Nlst6* dsRNA, 80% for the control group on media amended with dsGFP and 81% for controls maintained on untreated media. Relative *Nlst6* transcript levels in brachypterous females were down to about 40%, compared to ds*GFP* and untreated controls through seven days after adult emergence (Fig. 1).

### Dietary ds*Nlst6* led to reduced body weight

**Figure 2:**
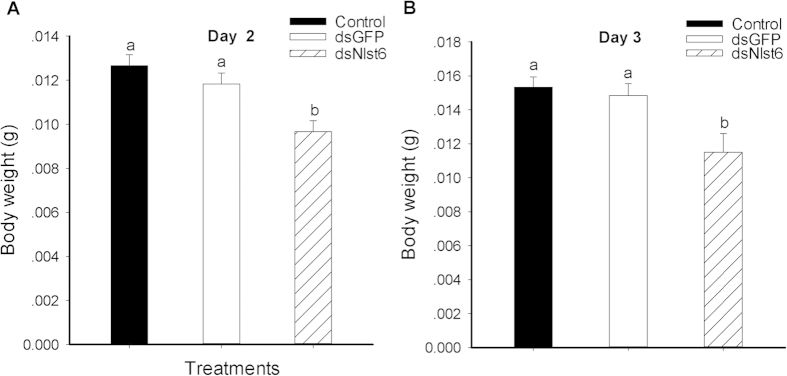
Effects of feeding-based dsRNA treatments on body weight of brachypterous females. Histogram bars show the means ± SE of six independent replicates, each with 5 females; histogram bars annotated with the same letters are not significantly different at a *p* < 0.05.

Dietary dsRNA treatments led to decreased female body weights, down by about 31% (3.0 mg) compared to untreated controls and by approximately 22% (2.1 mg) compared to dsGFP-treated controls at day 2 (Fig. 2A). At day 3, body weights of experimental females declined by 33% (3.8 mg) compared to untreated controls and by nearly 29% (3.3 mg) compared to dsGFP-treated controls (Fig. 2B).

### Dietary ds*Nlst6* led to reduced ovarian and fat body protein content

**Figure 3:**
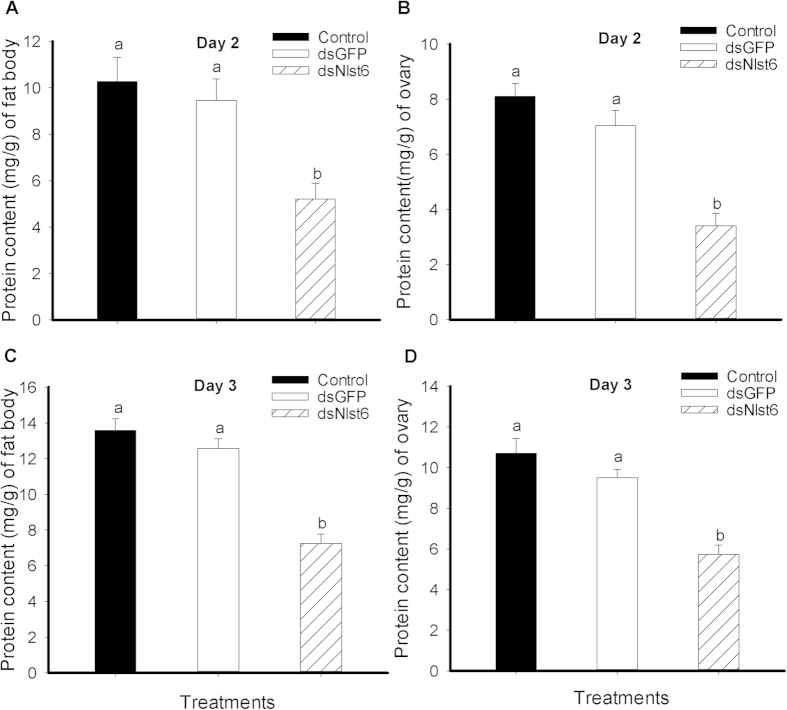
Effects of feeding-based dsRNA treatments on the protein content of brachypterous female fat bodies (Panels A & C) and ovaries (Panels B & D). Histogram bars report mean protein content (mg/g, n = 3 independent biological experiments) ± SE Histogram bars annotated with the same letter are not significantly different (t-test, *p *< 0.05).

Dietary ds*Nlst6* led to substantially reduced fat body protein content at day 2, down by 49% (by about 5.4 mg/g) and day 3 by 47% (by about 8.1 mg/g) (Fig. 3A-C). We recorded similar reductions in ovarian protein content at day 2, down by circa 58% (by about 2.5 mg/g) and day 3, reduced by about 46% (by 6.7 mg/g) (Fig. 3B–D).

### Dietary ds*Nlst6* led to reduced fecundity

**Figure 4:**
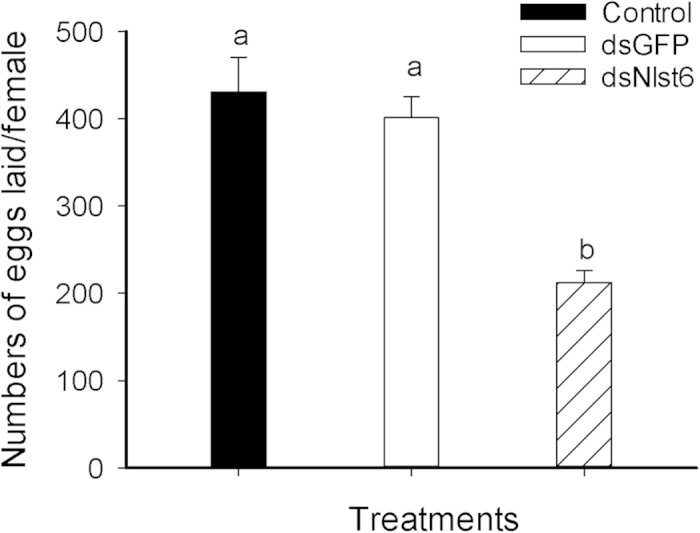
Dietary ds*Nlst6* treatments led to decreased egg laying (fifteen pairs were analyzed per group). The histogram bars report mean numbers of eggs laid/female ± SE of 15 replicates. Histogram bars annotated with the same letter are not significantly different (t-test, *p *< 0.05).

The ds*Nlst6* treatments led to reduced fecundity, expressed as numbers of eggs laid, down about 51%, from 430.8 eggs/female in controls to 212.3 eggs/female in experimental BPH (Fig. 4).

### The influence of dietary ds*Nlst6* on the duration of pre-oviposition and oviposition periods

**Figure 5:**
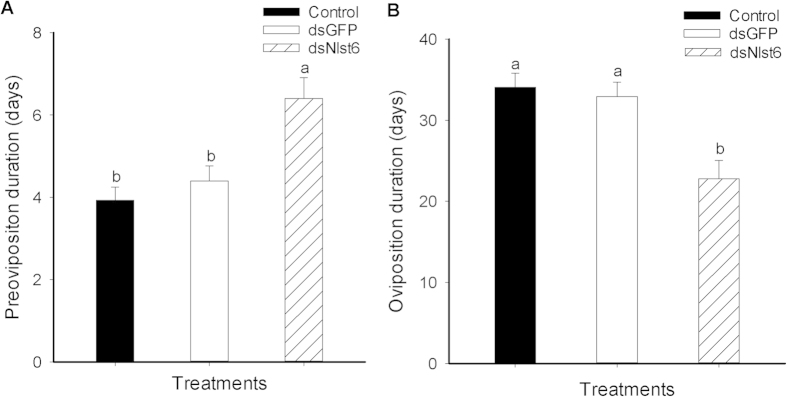
Effects of feeding-based ds*Nlst6* treatments on the duration of preoviposition period (Panel A) and oviposition period (Panel B) of brachypterous females. Histogram bars report numbers of days ± SE, n = 15 independent biological experiments; bars annotated with the same letter are not significantly different at a *p *< 0.05.

The pre-oviposition period is the time, in days, from adult emergence to the onset of egg-laying. The dietary ds*Nlst6* treatments increased this parameter from about 4 to over 6.4 days, compared to untreated controls (up circa 60%) and ds*GFP-*treated females (up about 45%; Fig. 5A). The oviposition period is the time, in days, of egg-laying, which was substantially reduced, by roughly 31 to 33% from about 33–34 to 22.8 days, compared to controls and dsGFP-treated BPH (Fig. 5B).

### The influence of dietary ds*Nlst6* on female longevity

**Figure 6:**
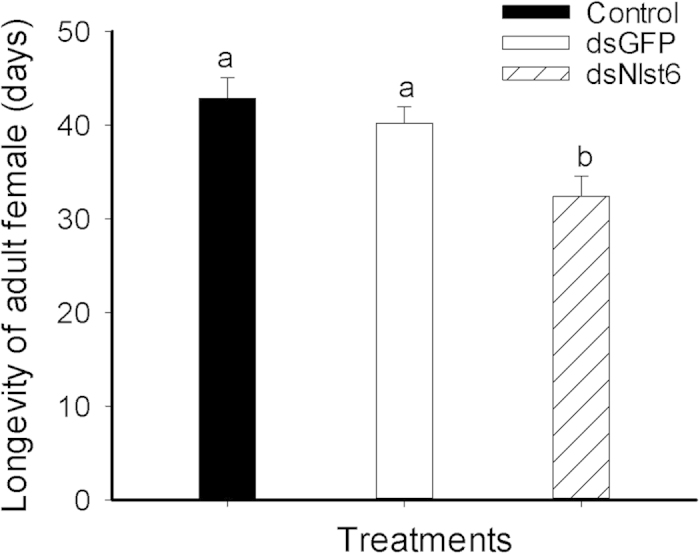
Dietary ds*Nlst6* treatments decreased female longevity. Histogram bars report longevity in days ± SE, n = 15 independent biological experiments; bars annotated with the same letter are not significantly different at *p *< 0.05.

Figure 6 shows the longevity of adult females exposed to dietary ds*Nlst6* was reduced compared to untreated controls (by around 21%) and ds*GFP-*treated controls (by about 19%), from about 40–41 days to about 32.4 days.

### The influence of dietary ds*Nlst6* on female fat body glucose content

**Figure 7:**
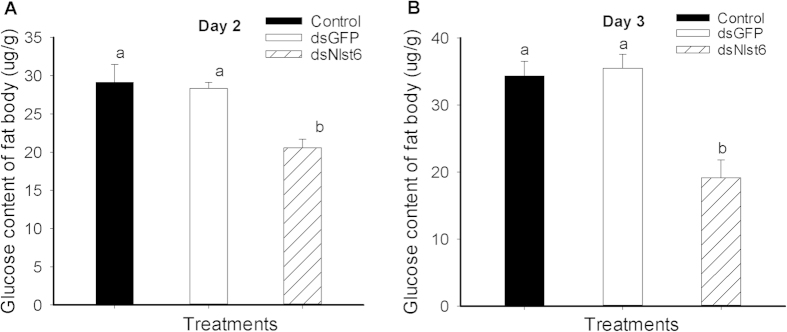
Effects of feeding-based dsRNA on the glucose content of brachypterous female fat bodies in day 2 (Panel A) and, separately, day 3 (Panel B) adults. Histogram bars report the mean glucose content (mg/g) ± SE, n = three independent biological experiments; bars annotated with the same letter are not significantly different at a *p* < 0.05.

Fat body glucose content in ds*Nlst6*-treated females was lower, compared to controls and ds*GFP*-treated controls, decreasing by 29%, from about 28 to 20 μg/g at day 2 (Fig. 7A) and by about 48% (nearly 18 μg/g) at day 3 (Fig. 7B) while no significant differences were found between the control and ds*GFP*-treated groups.

### Dietary ds*Nlst6* led to reduced expression of *Nlvg* mRNA

**Figure 8:**
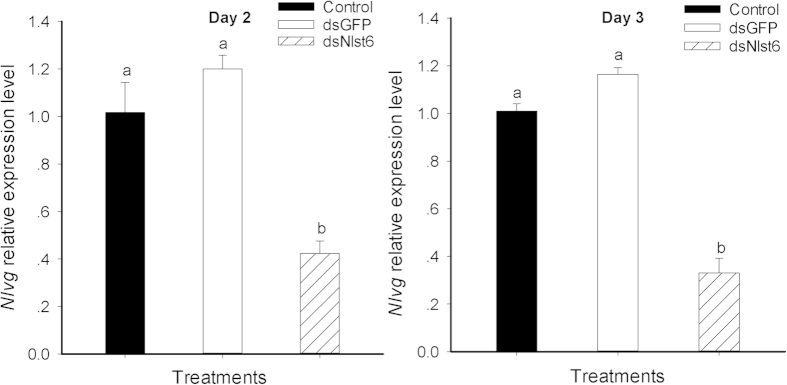
Dietary ds*Nlst6* treatments decreased *Nlvg* mRNA expression in day 2 (Panel A) and, separately, day 3 (Panel B) females. All values were normalized relative to β-actin transcript level. The data represent the mean values ± SE of three replicates. Histogram bars annotated with the same letter are not significantly different (t-test, *p* < 0.05). Each treatment and control experiment was repeated three times.

Figure 8 reports that dietary *dsNlst6* led to reduced expression of *Nlvg* in day 2 (down by 59%) and day 3 (down by 67%) females, compared to controls.

## Discussion

The data reported in this paper strongly support our hypothesis that *Nlst6* silencing lethally reduces BPH fitness in agroecosystems, seen in the following summary. First, dietary ds*Nlst6* treatments reduced expression of the sugar transporter transcript. Second, the dietary treatments led to decreased female body weight, lowered fat body and ovarian protein contents, diminished fecundity, increased pre-oviposition periods, reduced oviposition periods, shorter female longevity, decreased fat body glucose contents, and truncated expression of the BPH vitellogenin gene. Taken together, the uniformly deleterious changes in each of these biological performance parameters amount to a very potent argument that full expression of *Nlst6* is absolutely required to produce fully capable and fit adult BPHs. More generally, sugar transporters are essential features of successful feeding and energy metabolism in BPHs and likely all insect species.

*Nlst6* is expressed mainly in midgut, along with *Nlst*s 1, 4, 9, 12, 14 and 16^11^. Given that midgut expresses seven glucose transporters, the idea that silencing expression of one of the seven genes (by about 60%) could translate into marked deleterious changes at the whole-organism level may seem incongruous. We note that the parameters we measured were reduced to levels significantly, but not catastrophically, lower than control levels. Body weights were down by about 20–30%, ovarian and fat body protein and fecundity down about 50%, longevity down about 25%. It would seem that optimal operation of all seven transporter genes is necessary at the organismal level. We expect that dsRNA constructs that simultaneously silence two or more midgut *Nlst*s would exert greater impact on BPH biology. We analyzed ovaries and fat body with a focus on reproduction. The reduced protein and sugar contents in these tissues open questions about other tissues, including muscles. Specifically, would protein and sugar contents be similarly reduced on other tissues? Also, do the dietary dsRNA constructs operate solely at the midgut or do they operate systemically to influence sugar transport in individual tissues? While these issues are beyond the scope of this paper, we foresee research to address them in detail.

Body size or weight is, in some animals, a determinant of insect health^16^. Across butterfly superfamilies Papilionoidea and Hesperioidea, there is a correlation between body size and egg size in which larger eggs tend to develop into larger adults. Within a subset of butterfly species, fecundity is related to adult body size, although this can be influenced by local climate^17^. Mating with average-sized or heavy black-lyre leafroller females, *Cnephasia jactatana,* leads to more offspring, which have a higher probability of survival^18^. However, this broad generalization on the benefits of weight does not apply to all mating systems. For example, the reproductive efficacy of the pyralid moth, *Parapediasia teterrella,* is positively associated with low to average female weights, but is negatively associated with weights exceeding the average of the population^19^. Our data show that disruptions in sugar transport led to reduced female weights, which were attended by deleterious outcomes in several parameters of biological performance, particularly fecundity and longevity. In our view, the reduced weight of experimental females is a consequence, rather than cause, of the fitness declines we recorded.

The dietary *Nlst6* treatment led to reduced *Nlvg* mRNA expression levels. Exposure to some insecticides, deltamethrin and triazophos, for examples, similarly induces increases in *Nlvg* mRNA expression^20^ and promotes protein synthesis in adult female fat bodies and ovaries^21^. Alternatively, two other insecticides, chlorantraniliprole and indoxacarb, suppressed *Nlvg* mRNA expression, decreased fat body and ovarian protein content and attenuated the number of eggs laid^22^. These insecticides exert their actions through mechanisms unrelated to sugar transporter genes, however, they bring out the idea that the changes in fitness parameters that follow exposure to sub-lethal doses of some insecticides or ingestion of gene-silencing constructs can be quite similar. With respect to deploying dsRNA constructs at the greenhouse or field levels, we surmise that some of the effects of dsRNA can look, in terms of physiology and crop protection, like the effects of insecticides.

Previous investigations discussed relationships between yeast-like symbionts (YLS) and amino acid requirements in BPH^23^,^24^,^25^. Meeting essential amino acid requirements in BPH was associated with the abundance of YLS^23^. Artificial reduction of the symbiont abundance led to delayed growth and decreased survival rate, adult emergence rate, body weight, and fecundity^24^. Compared to controls, asymbiotic aphids, *Acyrthosiphon pisum* and BPHs grew more slowly, weighed less and contained less protein^25^. As to a physiological role, the YLS provide essential amino acids and possibly proteins required for host embryo formation and post-embryonic development^26^. The symbionts are essential to meeting nutritional amino acid needs for production of a healthy complement of proteins. Although we do not present data on the point, we infer the sugar transporter gene (*Nlst6*), mediates uptake of sugars necessary for host and symbiont maintenance and reproduction.

*Nlst6* silencing led to decreased protein content in the fat bodies and ovaries. The reduced protein contents can disrupt BPH biology, including production of yolk proteins. Vitellogenins are produced in fat bodies and the reduced fat body protein contents may impose a limit on the ability to produce vitellogenin, which can restrict ovarian development and, hence, reproduction.

The dietary ds*Nlst6* treatments led to decreased female fat body glucose as well as protein. Sugars are also accumulated in fat body cells as glycogen for long-term storage. We did not determine amounts of stored glycogen, and the impact of silencing *Nlst6* on glycogen stores remains to be determined. Lipid and soluble sugar contents in nymphs, and adults developed from nymphs feeding on rice plants treated with triazophos, was significantly increased compared with controls^27^. Our previous results showed that triazophos significantly stimulated fecundity of BPH females^20^. We speculated that the YLS populations within individual BPHs are somehow increased following exposure to triazophos at sub-lethal concentrations^20^. Our working hypothesis holds that dietary *Nlst6* led to nutrient deprivation in the symbionts, as well as in BPHs, which entrained a series of conditions. The deprivation led to reduced YLS populations, which led to reduced synthesis of essential amino acids, leading to reduced capability to biosynthesize proteins and, finally, to gaps in the BPH expressed proteome. This partially explains the reduced protein contents in fat bodies and ovaries.

A more complete explanation unfolds with recognition that reducing *Nlst6* expression led to restricting the global availability of nutrients within experimental BPHs. Cells respond to nutrient deprivation by inducing autophagy, a catabolic process that breaks down cytosolic components, including proteins and organelles to reallocate their component molecules toward necessary energy-generating metabolism. Once induced, however, autophagy is suppressed by TOR signaling, known in mammals and *Drosophila*^28^. Autophagy is thought to protect cells during nutrient deprivation. Hence, we speculate that protein catabolism during the autophagy response is partially responsible for reduced protein contents within fat body and ovarian cells.

BPH, and possibly other hemipterans, have 18 sugar transporter genes^11^. Some of them, *Nlst5, 8, 10, 14 and 16*, are substantially expressed in most tissues, including head, thorax, abdomen, midgut, ovary, testes, salivary gland, Malpighian tubules and fat body. Seven are expressed in the midgut, where they probably act in moving sugar from the midgut lumen to hemolymph circulation. *Nlst5*, 8, 10, 11, and 13 are expressed in the fat body, where they may operate in fat body sugar mobilization. Other *Nlst* genes are expressed during the embryonic stage, and facilitate sugar mobilization during embryonic development. Sugar transporters operate throughout the BPH lifecycle. Disruption of these transporters can have far-reaching effects on BPH populations. Recalling the very high mortality of second instar nymphs, which may lead to very efficacious field applications, we propose continued research into nutrient transporters in pest insects, which may yield new target genes for management of phloem-feeding pests through RNAi based transgenic plants.

## Materials and Methods

### Rice variety and culture

Rice (*Oryza sativa* L.) variety Ninjing 4 (japonica rice, commonly grown in Jiangu province) was used in all experiments. Seeds were sown outdoors in cement tanks (height 60 cm, width 100 cm, and length 200 cm) containing standard rice-growing soil. When seedlings reached the six-leaf stage, they were transplanted into 16 cm diameter plastic pots containing four hills per pot, three plants per hill and used for experiments at the tillering stage.

### Insect culture

BPHs were obtained from a laboratory population maintained in a greenhouse under our standard conditions (26 ± 2^o^C, with 70–80% humidity and a 16L:8D photoperiod at Yangzhou University. The insect colony was originally obtained from the China National Rice Research Institute (Hangzhou, China). Before the experiments started, the colony was allowed to reproduce for two generations in cement tanks (60 × 100 × 200 cm) under natural condition in Yangzhou.

### RNAi

We designed gene-specific ds*Nlst6* primers and amplified a 384-bp (672–1055 bp) *Nlst6* cDNA fragment using forward and reverse primers containing the T7 primer sequence at the 5′ ends (Table 1). The amplification program was 35 cycles of 95 °C for 40 s, 58 °C for 40 s and 72 °C for 1 min, with a final extension step of 72 °C for 10 min. The sequence was verified by sequencing (Invitrogen Company, Shanghai, China). We used the GFP gene (ACY56286; was provided by Zhang Chuan-xi, Institute of Insect Sciences, Zhejiang University) as control dsRNA and amplified a 688 bp fragment using primers listed in Table 1. We used the T7 RiboMAX ^TM^ Express RNAi System (Promega, Sunnyvale, CA) for dsRNA synthesis, following the Promega instructions. Sense and antisense dsRNAs generated in separate 20 μL total reaction volumes were annealed by mixing both transcription reactions and incubating at 70 °C for 10 min, and then cooling to room temperature over 20 min time period. A 2 μL RNase A solution (4 mg/ml) and 2 μL RNase-free DNase (1 u/μL) were added to the reaction tube and incubated in a 37 °C water bath for 30 min. The dsRNA was precipitated by adding 110 μL 95% ethanol and 4.4 μL 3 M sodium acetate (pH5.2), washed with 0.5 mL 70% ethanol and dried at room temperature. The dried product was dissolved in 50 μL nuclease-free water. The purified dsRNAs were quantified by spectroscopy. To deliver dsRNA into BPH, nymphs were reared on an artificial diet^29^, with some modifications to the rearing protocol, amended with dsRNA. Previous results indicated that dsRNA feeding led to rapid and significant reduction expression levels of BPH genes^30^. We used glass cylinders (15.0 × 2.5 cm diameter) as feeding chambers, using four concentrations, 0.125 μg/μl, 0.075 μg/μl, 0.05 μg/μl, and 0.025 μg/μl. The dsRNA solution (the final concentration, 0.05 μg/μl diet, was determined from the concentrations mentioned just above) was added to the artificial diet (20 μl), held between two layers of stretched Parafilm M membrane that were enclosed at the two open ends of the chamber (the diet capsule). The diet capsule was replaced every second day. The cylinders were covered with a piece of black cotton cloth, but the two ends with the artificial diet were exposed to light. Insects fed on the diets by puncturing the inner Parafilm M membrane of the diet capsule. All insects were transferred into chambers and maintained on artificial diets for one day before initiation of the assays. Twenty 3^rd^ instar individuals were transferred into each chamber, and three chambers were used to create three independent biological replicates. The rearing experiments were carried out in a humidified growth cabinet at 26 ± 2 °C, 90% RH and a 16L:8D photoperiod. Mortality was recorded every other day.

### The influence of dsRNA on biological performance parameters

We determined the effects of ds*Nlst6* treatments on several biological performance parameters. We exposed second instar nymphs to the dsRNA construct, which led to over 95% mortality. Ergo, third instar nymphs were transferred to diet capsules containing dsRNA-laced diet; at their fifth (final) instar (8 days), they were collected and a single nymph was transferred into a glass jar (12 cm high x 10 cm ) and reared on tillering rice plants under 26 ± 2 °C and 16L:8D. Eighty adult females were collected separately at days 2 and 3 after emergence, and fresh body weight, soluble fat body sugar content, ovary and fat body protein content, and *Nlst6* expression level were determined. We paired a single newly-emerged adult female with a male. Each pair (♂ × ♀) was maintained in a glass jar (diameter 10 cm, height 12 cm) with rice seedlings under our standard controlled conditions for oviposition. Fifteen copulating pairs were maintained to record duration of the pre-oviposition period, oviposition period, adult female longevity, and fecundity for each copulating pair. Rice stems were replaced daily during the pre-oviposition period, at two day intervals during the oviposition period and three day intervals during the female longevity period until the females died. The numbers of eggs laid on each rice stem was recorded under a light microscope. Eggs were scraped from the leaf sheaths and blades using a pin. Fecundity of 15 mated pairs was recorded as the average number of eggs produced by the mated female.

### Protein analysis

Protein was extracted from fat bodies and ovaries using a method similar to Gong *et al.*^31^ Individual adult females were dissected under a zoom-stereomicroscope (model XTL20, Beijing Tech Instrument Co., Ltd., Beijing, China) in a cooled petri dish. Ovaries and fat bodies of females were removed and placed in separate, pre-weighed, ice-cold centrifuge tubes and then re-weighed using a Mettler-Toledo electronic balance (EC100 model; 1/10,000 *g* sensitivity). A proportional amount of NaCl solution (0.4 M NaCl: 1M PMSF, v:v at a ratio of 20 ml NaCl solution to 1 g ovary or fat body) was added to the tube, homogenized on ice, and centrifuged at 16,000 × *g* at 4 °C for 20 min. The supernatant was collected after filtering the upper fat layer with glass fibers, placed at 4 °C overnight after adding ddH_2_O (1 supernatant: 10 ddH_2_O, v/v), and centrifuged again at 2,800 × *g* at 4 °C for 20 min. The protein sediment was dissolved with 1.5 ml pre-chilled 0.4 M NaCl solution after removing the supernatant.

We followed the procedure described in Brandford^32^ to measure protein content using Coomassie Brilliant Blue R250 (Shanghai Chemical Agent Co., Ltd., Shanghai, China). A standard curve was established based on a standard protein (bovine serum albumin, Shanghai Biochemistry Research Institute, Shanghai, China). The absorbance at 595 nm was determined in a UV755 B spectrometer (Shanghai Precision Instrument Co., Ltd., Shanghai, China). The protein content in the sample solution was calculated according to the standard curve

### Glucose analysis

Glucose levels were determined using the glucose oxidase method, following Sigma instructions (Sigma, St. Louis, MO). Two ml of 20 mM phosphate buffer (PBS, pH 5.8) were added to 20 mg of whole insect ovaries and fat bodies, and then homogenized at 0 °C. The sample solution was centrifuged at 11,200 × *g* for 10 min at 4 °C. Fifty μl of 0.6 mol.L^−1^ perchloric acid were added into 50 μl of the supernatant to remove the protein in solution. After centrifugation at 1,008 × *g* for 5 min at 4 °C, 450 μl of 0.2 mol. L^−1^ sodium phosphate buffer (pH 7.4) was added into 50 μl of the supernatant. Two hundred μl of chromogen reagent (Covance, New Jersey, USA) and 150 μl of glucose oxidase were added to 50 μl of the sample solution, then incubated at 37 °C for 5 min. The absorbance at 625 nm was determined with a UV755B spectrometer (Shanghai Precision Instrument Co., Ltd., Shanghai, China). The glucose content in the sample solution was calculated based on a standard curve.

### Body weights

Five females were used as a replicate at day 2 and day 3. Five adult females were placed in pre-weighed centrifuge tubes and then weighed using a Mettler-Toledo electronic balance (EC100 model; 1/10,000 *g* sensitivity). Each treatment and each control experiment was replicated six times.

### qPCR analysis

We isolated total RNA from the five newly-emerged females, using a SV Total Isolation System Kit (model Z3100, Promega Corporation, Madison, WI, USA). First-strand cDNA was synthesized according to the instructions supplied with the PrimeScript RT reagent Kit (TaKaRa Biotechnology (Dalian) Co., Ltd). First-strand cDNA was synthesized in a 10 μl reaction volume containing 0.5 μg of RNA, 0.5 μl of PrimeScript RT enzyme mix I, 0.5 μl of Oligo dT primer (50 μM), 2 μl of random hexamers (100 μM), 2 μl 5 × PrimeScript Buffer (for real time-PCR) and RNase–free dH_2_O up to a final volume of 10 μl. The cDNA was reverse transcribed using the following program: 37 °C for 15 min, 85 °C for 5 s and 4 °C for 5 min.

We isolated total RNA from the dsRNA-treated and un-dsRNA (control) females, using a SV Total Isolation System Kit (model Z3100, Promega Corporation, Madison, WI, USA). Portions (2 μl) of the synthesized first-strand cDNA (above synthesis) were amplified by qPCR in 20 μl reaction mixtures using a CFX96 real-time PCR system (Bio-Rad Co. Ltd., California, USA) with the following procedure: 94 °C for 2 min, followed by 40 cycles of 94 °C for 5 s, 61.8 °C (*Nlst6*) or 58 °C (*Nlvg*) for 30 s, and 72 °C for 30 s. The differences in the T_m_ values of *Nlst6* or *Nlvg* was 61.8 °C or 58 °C, other procedures were the same. *Nlst6* (AB549999) or *Nlvg* (AB353856) mRNA levels were quantified in relation to constitutive actin-1 expression (EU179846). Primers used for qPCR analysis are listed in Table 1. After amplification, a melting curve analysis was performed in triplicate and the results were averaged. The values were calculated using three independent biological samples and the 2 ^−△△CT^ method^33^ used for the analysis of relative *Nlst6* expression level.

### Statistical analysis

The results presented in figures are expressed as the means ± S.E. DPS 7.5 software was used to perform t-tests to identify significant differences at a 95% confidence level (*p*  0.05)^34^. Biological parameter data were analyzed using an analysis of variance (ANOVA) with one factor and statistical data are presented in Table 2.

## Additional Information

**How to cite this article**: Ge, L.-Q. *et al.* Silencing a sugar transporter gene reduces growth and fecundity in the brown planthopper, *Nilaparvata lugens* (Stål) (Hemiptera: Delphacidae). *Sci. Rep.* **5**, 12194; doi: 10.1038/srep12194 (2015).

## Figures and Tables

**Table 1:** PCR primers used in this study.

Primers	Primer sequence	Product size
For real-time PCR		
Q-Nlst6-F	CATCGGAGAAATCGCTGAG	179 bp
Q-Nlst6-R	CTGGCATGAAGAAGAACAAA	
Q-Nlvg-F	GTGGCTCGTTCAAGGTTATGG	200 bp
Q-Nlvg-R	GCAATCTCTGGGTGCTGTTG	
Actin-F	TGCGTGACATCAAGGAGAAGC	186 bp
Actin-R	CCATACCCAAGAAGGAAGGCT	
For dsRNA synthesis		
Nlst6-F	GGAGAAATCGCTGAGGAC	384 bp
Nlst6-R	GCCGCAACTGAGGTAAAG	

**Table 2:** Statistical analyses of biological parameter data.

Experiment	Significant?	F statistic
Survival	No	*F* = 0.19, df = 2, 42, *P* = 0.82
Body weight, day 2	Yes	*F* = 10.1, df = 2, 15, *P* = 0.0017
Body weight, day 3	Yes	*F* = 6.3, df = 2, ,15, P = 0.0101
Fat body protein, day 2	Yes	*F* = 9.2, df = 2, 6, *P* = 0.0148
Fat body protein, day 3	Yes	*F* = 35.2, df = 2, 6, *P* = 0.0005
Ovarian protein, day 2	Yes	*F* = 24.8, df = 2, 6, *P* = 0.0013
Ovarian protein, day 3	Yes	*F* = 22.8, df = 2, 6, *P* = 0.0016
Fecundity, as eggs laid	Yes	*F* = 17.9, df = 2, 42, *P* = 0.0001
Pre-oviposition period	Yes	*F* = 10.6, df = 2, 42, *P* = 0.0002
Oviposition period	Yes	*F* = 10.2, df = 2, 42, *P* = 0.0002
Female longevity	Yes	*F* = 7.1, df = 2, 42, *P* = 0.0023
Fat body glucose, day 2	Yes	*F* = 8.1, df = 2, 6, *P* = 0.0196
Fat body glucose, day 3	Yes	*F* = 15.8, df = 2, 6, *P* = 0.0045
*Nlvg* expression, day 2	Yes	*F* = 104.6, df = 2, 6, *P* = 0.0001
*Nlvg* expression, day 3	Yes	F = 28.6, df = 2, 6, *P* = 0.0009
